# Women's Free-text Comments on their Quality of Life: An Exploratory Analysis from the UK Standardisation of Breast Radiotherapy (START) Trials for Early Breast Cancer

**DOI:** 10.1016/j.clon.2018.03.007

**Published:** 2018-07

**Authors:** J. Mills, J.S. Haviland, C. Moynihan, J.M. Bliss, P. Hopwood, the START Trial Management Group

**Affiliations:** ∗ ICR-Clinical Trials and Statistics Unit (ICR-CTSU), Division of Clinical Studies, The Institute of Cancer Research, London UK; † Department of Genetics & Oncology, The Institute of Cancer Research, London UK

**Keywords:** Breast cancer, clinical trials, contextual factors, patients'free-text comments, quality of life, radiotherapy

## Abstract

**Aims:**

Exploratory analysis of patients' unsolicited written comments in the first 2 years of the Standardisation of Breast Radiotherapy (START) trial quality of life study highlighted a potential effect of non-treatment-related problems on the ratings and interpretation of patient self-reported questionnaires. At 5 years of follow-up all eligible subjects were invited to write comments to further explore these findings.

**Materials and methods:**

Using inductive qualitative methods informed by the exploratory analysis, comments were allocated to relevant themes. Key patient-reported outcome measures (PROMs), clinical and demographic factors were collated for patients who did and did not comment at 5 years and comparisons between the groups explored.

**Results:**

Of 2208 women completing baseline PROMs, 482 proffered comments from 0 to 24 months, forming nine distinct themes, including chronic conditions, life events and psychosocial concerns. At 5 years, 1041/1727 (60.3%) women contributed comments, of whom 500 randomly selected participants formed the sample for analysis. Findings revealed comorbidity, impaired physical functioning and psychosocial problems as key themes, with prevalent adverse effects from local and systemic treatments. Eight new themes emerged at 5 years, including ageing, concerns about future cancer and positive aspects of care. Women commenting were better educated, slightly older and more likely to have had chemotherapy compared with non-commenters. They had significantly worse PROM scores for global health and key quality of life domains relevant to the difficulties they revealed.

**Conclusions:**

Difficult personal circumstances and other health concerns affected many women's PROM ratings at 5 years of follow-up, in addition to ongoing cancer treatment effects. Greater attention to multiple sources of distress and adversity could facilitate personalised care and aid interpretation of PROMs.

## Introduction

The findings of the Standardisation of Breast Radiotherapy (START) trial's quality of life substudy [1] provided valuable information for patients and clinical teams about beneficial and unfavourable effects of the radiotherapy treatment groups under comparison, as an aid to future decision-making and clinical care provision. START tested a widely used dose regimen (40 Gy in 15 fractions) and two test schedules of hypofractionated radiotherapy (fractions >2.0 Gy) against the international standard of 50 Gy in 25 fractions, in terms of local tumour control and late normal tissue effects. Findings from patients' ratings strengthened the evidence in support of the clinical findings in favour of hypofractionated regimens [2], [3], which influenced clinical breast radiotherapy practice [4]. The quality of life findings were derived from standardised measures designed within a biomedical framework, which included questions relating to protocol-specific radiotherapy effects that helped distinguish between the regimens.

Such measures are very effective in supporting key end points in clinical trials and cover a range of largely biomedical domains to facilitate multidimensional comparisons between treatment arms and have contributed to clinical improvement. However, they are not designed to encompass non-trial circumstances or individual experiences and so may not inform individual care. There has been extensive psychosocial research detailing the multiple and complex effects of breast cancer and its treatment [5], [6], [7], [8], [9], [10], [11], [12], [13], [14], but to date there has been little opportunity for patients to express the meaning or relevance of non-breast cancer symptoms, psycho-social problems or functional limitations in the context of a clinical trial [5], [15]. However, it is expected that randomisation will eliminate any bias due to individual circumstances for treatment comparisons in the trial setting.

Unexpectedly, during the first 2 years of quality of life data collection in the START trials, 22% of women wrote unsolicited comments at least once, or enclosed letters, when returning their quality of life booklets. These women frequently wanted to ‘explain’ that their responses to specific questionnaire items or subscales reflected the effects of other personal problems, life events or health issues rather than breast cancer or its treatment. Some women thought there should be space for such reporting: ‘Completing the questionnaire I thought there should be a question about whether there are any factors/worries in your daily life that affect your answers’. These patients also expressed concern that if contextual factors were sufficient to influence their questionnaire ratings they could be misattributed to effects of cancer treatments. The potential value of these comments in raising awareness of contextual problems in the clinical setting and of their possible influence on quality of life ratings warranted further exploration. We therefore conducted a qualitative study of the comments proffered up to 2 years and a summary of the sample composition, analysis and findings is presented as supplementary data in Appendix A. These were found to endorse the importance to quality of life of comorbidity and other contextual factors, not captured by the quality of life measures, and the potential for misattribution of ratings to breast cancer outcomes. If generalised, these contextual factors could lead to inferior quality of life outcomes for long-term survivors in whom the interplay of contextual factors, life stress and ageing may impede adjustment and be detrimental to coping, decision-making and ongoing self-management [16], [17], [18], [19].

Following on from this, and given the relatively small sample of women who proffered comments early on in the trial, it was decided to invite comments from all women in the START trials completing patient-reported outcome measures (PROMS) at the 5 year assessment. The aims were: (i) to retrospectively explore reported health concerns and adverse contextual factors and see if they endorsed the proffered comments, and (ii) to examine possible associations between quality of life scores derived from the quantitative questionnaire items and patients' reported health concerns and other adverse contextual factors.

## Materials and Methods

Full details of the UK START trials and quality of life substudy have been published separately [1], [2], [3]. The START trials were registered as an International Standard Randomised Controlled Trial, number ISRCTN59368779. Patients were recruited to the quality of life study from 31 of 35 radiotherapy centres in the UK between 1998 and 2002 and the main quality of life outcomes were published in 2010 [1]. Ethical approval was obtained from the South Thames Multi-Research Ethics Committee to request additional written comments from all patients completing the 5 year quality of life follow-up assessment; local ethics committees of all participating centres also gave approval. A blank page in the PROMS booklet was included and a patient information letter invited participants to report any health problems or events that they thought might influence the answers they gave in their PROMS booklet (see Appendix B for full text).

The quality of life booklets comprised the European Organization for Research and Treatment of Cancer (EORTC) QLQ-C30 core questionnaire [20], EORTC QLQ-BR-23 breast cancer-specific module [21], Hospital Anxiety and Depression Scale [22], Body Image Scale [23] and a health economics evaluation [24] for completion at home. The trials office at the Institute of Cancer Research (ICR) first checked the individual's current health status with their hospital team or family doctor before sending questionnaires. Prompts were sent for non-return of questionnaires by letter or telephone 3 weeks after mailing. At 5 years, all pages with comments in the quality of life booklets were logged on the quality of life study database.

The number of comments received on the 5 year questionnaires was too large to analyse using the entire written records and so comments from a random sample of 500 patients were used for the analysis, which followed a constant comparative methodology [25], as described for the proffered comments (Appendix A). Thus, for each patient commenting, each written comment was allocated to an appropriate theme: initially all nine themes created from the proffered comments analyses were used (Appendix A). Additional themes were formed and labelled, for comments that had not previously been submitted. All decisions ascribing comments to ‘new’ themes were made jointly by at least three coders. Where there was difficulty allocating a theme, a consensus decision was made.

### Statistical Methods

Descriptive analyses compared demographic and clinical characteristics, and key quality of life scores between women who did and did not provide comments at 5 years. Quality of life subscale scores at 5 years were calculated as specified in the EORTC scoring manual [26].

A secondary analysis compared quality of life scores in three key domains (global health/quality of life, physical and emotional functioning) and two symptom items (BR23 ‘hot flushes’ and ‘worry about future health’) for women commenting on an associated theme with women who commented on different themes, to investigate associations between themes and corresponding quality of life scores. EORTC subscales were selected that were considered most likely to reflect differences between patients commenting or not commenting on key areas of concern, based on exploration of data from the proffered comments. For graphical presentation, the EORTC subscales were arbitrarily grouped into the following categories 0–40, 41–60, 61–80, 81–100, as the distributions of scores were highly skewed.

Differences were tested using either the *t*-test or Mann–Whitney test for continuous variables or the χ^2^ test or χ^2^ test for trend for categorical variables, as appropriate.

## Results

At 5 years, 91.2% (1728/1893) of eligible women completed quality of life questionnaire booklets and 60.2% (1041/1728) provided written comments; of these, 275/1041 (26.4%) had also proffered comments between 0 and 24 months. Four patients in the random sample selected for this analysis were excluded as their comments indicated only administrative issues. There were no clear differences between the randomised schedules in the proportion of women providing comments at 5 years. A comparison of the characteristics of women commenting or not commenting at 5 years (Table 1) shows that ‘commenters’ were slightly older and better educated than ‘non-commenters’; they were more likely to have received adjuvant chemotherapy and had significantly poorer quality of life across all EORTC functional subscales, and worse fatigue and pain symptom scores.

**Table 1:** Characteristics of women who wrote comments at 5 years and women who did not

	Women who commented at 5 years) *n* = 1041 (%)	Women who returned 5 year form but who did not comment *n* = 687 (%)	*P*-value
Age at baseline (years): mean (standard deviation) [range]	57.4 (10.0) [27–85]	56.2 (9.8) [28–82]	0.021∗
Highest level of education achieved§			<0.001†
None	326/981 (33.2)	284/624 (45.5)	
School certificate/O level/GCSE/NVQ or equivalent	275/981 (28.0)	185/624 (29.6)	
A level/HND or equivalent	60/981 (6.1)	30/624 (4.8)	
Degree, postgraduate or professional qualification	282/981 (28.7)	97/624 (15.5)	
Unknown – not completed on form	38/981 (3.9)	28/624 (4.5)	
Type of surgery			0.105‡
Breast-conserving surgery	906 (87.0)	578 (84.1)	
Mastectomy	135 (13.0)	109 (15.9)	
Chemotherapy			0.025‡
No	746 (71.7)	454 (66.1)	
Yes	295 (28.3)	229 (33.3)	
Unknown	0	4 (0.6)	
Tamoxifen			0.138‡
No	161 (15.5)	125 (18.2)	
Yes	880 (84.5)	558 (81.2)	
Unknown	0	4 (0.6)	
EORTC QLQ-C30 subscale scores at 5 years||			
Global health/quality of life			<0.001†
0–40	76 (7.3)	34 (4.9)	
41–60	204 (19.6)	102 (14.8)	
61–80	267 (25.6)	167 (24.3)	
81–100	489 (47.0)	379 (55.2)	
Unknown	5 (0.5)	5 (0.7)	
Physical functioning			<0.001†
0–40	41 (3.9)	19 (2.8)	
41–60	105 (10.1)	43 (6.3)	
61–80	228 (21.9)	115 (16.7)	
81–100	666 (64.0)	508 (73.9)	
Unknown	1 (0.1)	2 (0.3)	
Emotional functioning			0.011†
0–40	46 (4.4)	32 (4.7)	
41–60	129 (12.4)	53 (7.7)	
61–80	284 (27.3)	172 (25.0)	
81–100	579 (55.6)	424 (61.7)	
Unknown	3 (0.3)	6 (0.9)	
Role functioning			<0.001†
0–40	103 (9.9)	33 (4.8)	
41–60	46 (4.4)	18 (2.6)	
61–80	183 (17.6)	79 (11.5)	
81–100	705 (67.7)	552 (80.3)	
Unknown	4 (0.4)	5 (0.7)	
Social functioning			<0.001†
0–40	59 (5.7)	22 (3.2)	
41–60	41 (3.9)	9 (1.3)	
61–80	136 (13.1)	63 (9.2)	
81–100	802 (77.0)	588 (85.6)	
Unknown	3 (0.3)	5 (0.7)	
Cognitive functioning			0.009†
0–40	44 (4.2)	22 (3.2)	
41–60	67 (6.4)	28 (4.1)	
61–80	200 (19.2)	117 (17.0)	
81–100	727 (69.8)	515 (75.0)	
Unknown	3 (0.3)	5 (0.7)	
Fatigue symptoms			0.001†
0–40	793 (76.2)	578 (84.1)	
41–60	167 (16.0)	67 (9.8)	
61–80	59 (5.7)	30 (4.4)	
81–100	21 (2.0)	10 (1.5)	
Unknown	1 (0.1)	2 (0.3)	
Pain symptoms			<0.001†
0–40	859 (82.5)	618 (90.0)	
41–60	71 (6.8)	34 (4.9)	
61–80	70 (6.7)	17 (2.5)	
81–100	40 (3.8)	15 (2.2)	
Unknown	1 (0.1)	3 (0.4)	

Unknown categories excluded from significance tests.

∗ *t*-test.

† χ^2^ test for trend.

‡ χ^2^ test.

§ Education data collected at 1 year after randomisation, so not available for all participants.

|| EORTC QLQ-C30 scores range from 0 to 100: higher scores indicate better functioning but worse symptoms.

**Fig 1:**
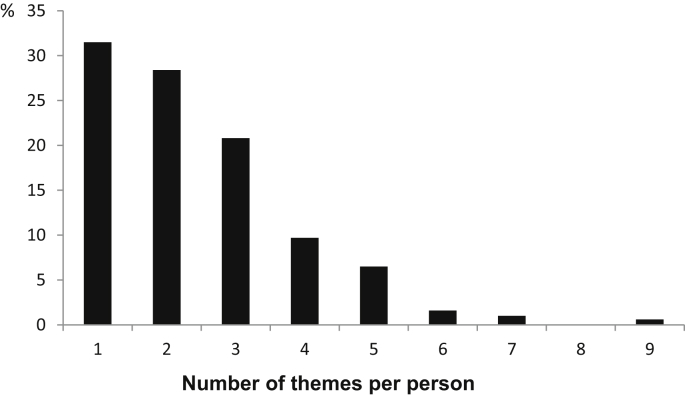
Number of themes identified per person from the random sample of 500 women commenting at 5 years.

Seventeen themes emerged from the 5 year comments (see Table 2 and Appendix B), including nine derived from the unsolicited comments (see Table A2 in Appendix A). The number of themes reported per person ranged from one to nine (Figure 1).

**Table 2:** Themes derived from women's invited comments at 5 years, showing most frequent component items, for the random sample of women who commented

**Negative comments**	Number (% of 496) of women reporting theme at 5 years
*Chronic medical problems including physical functioning:* Asthma, breathlessness, diabetes, heart disease, hypertension, chronic conditions, e.g. multiple sclerosis, migraine, Parkinson's disease, rheumatoid and osteoarthritis, fractures or falls, back or joint pain, fibromyalgia, muscle pain or injury, mobility problems	170 (34.3)
*Breast and related problems:* Breast pain, arm lymphoedema, cancer recurrence in the breast, breast abnormalities, radiotherapy effects to the breast, body image	129 (26.0)
*Systemic treatment side-effects:* Tamoxifen side-effects, hot sweats, weight gain, sexual problems	108 (21.8)
*Surgery (excluding breast cancer-related surgery) and hospital admissions* Gynaecological, dental, or other surgery, orthopaedic, falls	28 (5.6)
*Acute or transient health problems:* Colds, viral infections, ‘stomach upset’	47 (9.5)
*Psychological problems:* Depression or taking antidepressant medication, history of mental illness (e.g. schizophrenia) anxiety disorders, panic attacks, stress	89 (17.9)
*Life events or family problems:* Bereavement, husband's illness, house move, relationship problem	43 (8.7)
*Job problems:* Redundancy, early retirement, job loss and other job-related issues	10 (2.0)
*Other cancers:* Any cancer other than second primary breast cancer/recurrence/metastasis	12 (2.4)
*Aspects of care:* Problems with diagnosis, treatment or follow-up, general practitioner or specialist	17 (3.4)
*Family history of cancer:* Family members with cancer, genetic risk	14 (2.8)
*Effects of aging:* Older age affecting recovery, uncertain symptom attribution, e.g. tiredness	40 (8.1)
*Future concerns:* Cancer-related worries, fear of cancer recurrence	55 (11.1)
**Positive comments**	
*Good aspects of care:* Gratitude to hospital staff and trials unit, felt well supported	149 (30.0)
*Good recovery from cancer:* Feeling back to normal, positive attitude	80 (16.1)
*Personal support:* Support from friends, family, general practitioner, religion	27 (5.4)
*Positive life events:* e.g. grandchildren, family births, marriage	16 (3.2)

Percentages add up to >100% as some women have commented in more than one category.

The ‘chronic medical and physical functioning’ themes were combined at 5 years due to frequent overlap of reported conditions; this was the most frequently used theme (34.3% of women commented) compared with 26% reporting local breast-related problems and 21.8% commenting on systemic treatment-related problems. In contrast to earlier proffered comments, these local and systemic treatment concerns were expectedly more prevalent at 5 years. The personal and individual impact of treatment-related problems varied widely, highlighting their adverse impact on wellbeing, bodily changes, social activity, sex life and satisfaction.

One in six women reported current or chronic mental health difficulties, usually unrelated to cancer, whereas job problems, life events and family problems were less prevalent at 5 years than earlier in the trial. New themes included concerns about a cancer family history (reported by only 2.8%), ageing effects (8.1%) or concerns about the future (11%), especially fears of recurrence. Both dissatisfaction and satisfaction with medical and hospital care were expressed. Overall, two thirds of all written comments were negative or expressed difficulties and concerns. However, at this final 5 year assessment point in the START trials, 30% of written comments were brief remarks of gratitude for positive aspects of care and appreciation of trial participation; others reflected the value of personal support, positive life events and of making a good recovery (16.1%) (examples from all the themes at 5 years can be found in Appendix B).

**Fig 2:**
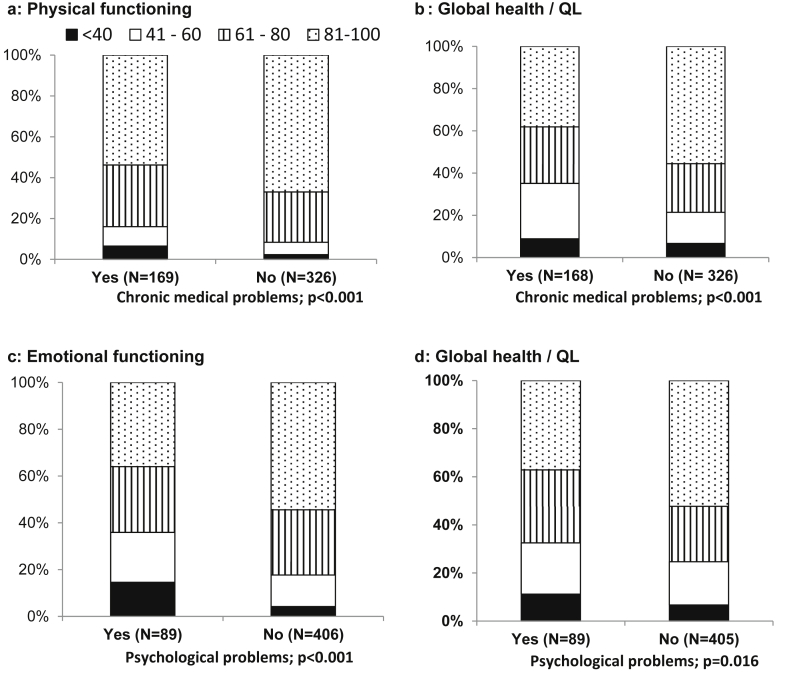
Specificity of themes derived from comments provided at 5 years with corresponding 5 year European Organization for Research and Treatment of Cancer (EORTC) QLQ-C30 subscale scores. Scores range from 0 to 100: higher scores indicate better functioning and global health Denominators vary due to missing data for some quality of life subscales. (a) Physical functioning subscale scores for women commenting/not commenting about chronic medical problems. (b) Global health/quality of life subscale scores for women commenting/not commenting about chronic medical problems. (c) Emotional functioning subscale scores for women commenting/not commenting about psychological problems. (d) Global health/quality of life subscale scores for women commenting/not commenting about psychological problems.

Comparing quantitative quality of life ratings between groups of women commenting on specific areas of concern, physical functioning and global health/quality of life scores were significantly worse for women who commented in the ‘chronic medical and physical functioning’ theme compared with women who had commented in different themes (Figure 2a, b). Similar quality of life differences for emotional functioning and global health/quality of life were found for women commenting versus those not commenting in the ‘psychological problems’ theme (Figure 2c, d). Furthermore, significantly more women (34%) commenting on systemic treatment side-effects rated their ‘hot flushes’ in the BR23 subscale as ‘quite a bit’ or ‘very much’ compared with 24.8% of ‘non-commenters’ on this item (*P* = 0.018). Scores on the BR23 ‘future perspective’ item were significantly worse for those commenting on their personal fear of recurrence (45.3% responded ‘quite a bit’ or ‘very much’) compared with women who did not comment in this theme (31.6% responded ‘quite a bit’ or very much’; *P* = 0.016).

## Discussion

Women's free-text comments in the START trials revealed many adverse personal circumstances at 5 years of follow-up as well as current and chronic health and psychosocial difficulties. For some women, breast cancer was not the only – or necessarily the main – determinant of their quality of life. For others, ongoing or late effects of multimodal breast cancer treatment significantly impaired their wellbeing. Overall, these comments endorsed and extended the proffered comments made by women earlier in the trial. They provide a broad, explanatory dimension to their quality of life ratings and highlight negative effects of comorbidity, life events and adverse psychosocial problems on many individual experiences of cancer and quality of life outcomes. These insights from a national radiotherapy trial setting are novel and informative but also resonate with many issues described by others, using examples from clinical research and practice and patients' testimonies [6], [7]. From these, wide-ranging contextual factors were uncovered that were considered critical to understanding patients' resources and experience of cancer.

We confirmed a significant negative impact on quality of life ratings for ‘commenters’ compared with ‘non-commenters’ over all functional domains, as well as specificity of the effect of particular themes on related quality of life domains. Comments on psychological problems often referred to specific reasons for their quality of life ratings, such as chronic mental health, family problems and general stress rather than breast cancer, adding informative value to an earlier assessment of psychological problems in the START trials [27]. Adverse effects of local and systemic breast cancer treatment effects were expressed by women in their comments in addition to completing quality of life questions in these key areas, adding personal details of the impact of these treatment effects, such as on body image and sexuality. Comments about chronic or disabling conditions and comorbidities were expectedly frequent in an ageing population and reflect evidence of both incident and chronic disease in survivors [13]. These conditions are of concern as they can lead to an inferior prognosis and worse disease outcomes [13], [19], [28], as well as indicating supportive care needs.

Comments about fear of recurrence and existential concerns were the most prevalent in the new themes at 5 years and have been highlighted as a key problem in recent research [29], [30]; other new issues reported may reflect women's increased awareness of genetic aspects, second cancers and the effects of ageing during survivorship. Expressions of positive outcomes and praise for health care and trial participation showed good supportive care in the trials setting and far outweighed criticisms.

### What are the Implications for Future Quality of Life Assessment?

Many issues raised in the written comments were not covered by the PROMS and this calls into question the scope and interpretation of quality of life data in future radiotherapy trials. However, the strength of these measures in the START trials was in determining selected biomedical outcomes from thousands of patients' ratings to determine differences between the treatment regimens, enhancing the clinical findings; other studies have found an association of PROMS with improved supportive care and patient satisfaction [31]. However, these questionnaires are not designed to differentiate between breast cancer-related and more general health or contextual problems. Concerns have been raised about the selected agenda of quality of life measures [15], which limit the discovery of additional influences on wellbeing [7]. It has been suggested that the assessment in clinical trials needs to be broadened to include environmental, economic, medical and social factors [6], [7], [15], [32]. Moreover, with few exceptions [33], symptoms are reported in PROMS in terms of their occurrence and severity over time rather than the distress or disruption caused to daily life.

### What is the Value of Self-reported Comments?

Women's comments reveal the experience of cancer and the impact of adverse events and day to day difficulties, as well as the interrelatedness of health and personal or social circumstances. For example, losing a job after diagnosis can lead to developing depression. However, distinctions between the contribution of treatment and other causes (comorbidity, physiological) to reported symptoms can still be difficult to determine. The value of this additional information extends beyond the randomised comparisons. However, can patients' free-text comments continue to make a valid contribution? Their utility has been endorsed in a large exploratory study of patients' views of cancer care [34] in which the potential value of free-text comments was described as ‘illuminating’ when highlighting potential causes for some inferior outcomes in a survey of mixed cancer patients [35]. Novel ways of analysis of these data have also been described [36]. However, free-text comments usually require a time-consuming methodology and analysis that is likely to preclude frequent use in large studies. Currently free-text comments are being invited in two further radiotherapy trials run by the ICR in order to explore earlier stages of treatment and follow-up for which adequate data were not available in the START trials.

In support of our findings, we found no significant difference between the different treatment schedules of the START trials in terms of frequencies of comments and can therefore be confident that there was no specific bias to the quality of life results on that basis. The quality of life sample gave good representation with respect to age and geographic area for breast cancer populations [37]. Using a constant comparative methodology enabled us to describe comments on the diverse social and medical context of women's breast cancer experience by creating distinct themes, but not to speculate on whether these women would be similar to age-matched population samples. We do not know if women commenting also reported and discussed their concerns with health professionals or received helpful interventions.

Considering potential confounders in our study, we recognise that women who chose not to comment may have felt non-cancer problems were irrelevant or inappropriate. Their apparent enhanced quality of life may reflect more adaptive coping, favourable resilience or adjustment to adversity. Beneficial effects of existing social care, psychological or medical intervention may also play a part. Women commenting were better educated and slightly older, which may have enhanced response rates, while those receiving chemotherapy perhaps felt more impetus to report related problems.

In conclusion, this novel opportunity to synthesise quantitative and qualitative data in the START trials provides a broader understanding of the cancer experience and the influence of acute and chronic contextual problems. Awareness of our findings should help clinical teams to address the wider effects of contextual difficulties, comorbidity and late effects of treatment, and stimulate thought about the interpretation and future development of PROMs. Many women accept trial participation for altruistic reasons [38] and their feedback should help inform the provision of individualised supportive care for future patients.

## Appendix A Supplementary data

mmc1
