# Supplementary material for: Women's Free-text Comments on their Quality of Life: An Exploratory Analysis from the UK Standardisation of Breast Radiotherapy (START) Trials for Early Breast Cancer
Source: Clin Oncol (R Coll Radiol). 2018 Jul;30(7):433–41. doi: 10.1016/j.clon.2018.03.007 (PMC6005815; doi:10.1016/j.clon.2018.03.007)
Supplement: mmc1 [file mmc1.docx]

**Supplementary Material**

**Appendix A: A preliminary exploration of proffered comments received between 0 and 24 months in the Quality of Life substudy in the START trials**

**Methods**

Unsolicited letters or comments written in the QL booklets received between 0 and 24 months in the START trials quality of Life substudy were abstracted and typed verbatim for each patient (identified by their study ID) for each time point, in order to explore their content in more detail.

An inductive qualitative methodology described by Pope (2000) was used to create themes for collating comparable comments. The initial analysis of unsolicited comments was carried out by 2 coders (JM,PH), with overall guidance from an expert qualitative researcher (CM). Each comment was allocated to an appropriate theme created by the first author (JM) and reviewed by a second researcher (PH), A constant comparative method was used, where verbatim reports were compared both within and between participants’ responses, to identify independent themes from their content. Where a theme was unclear, the classification was reviewed jointly until agreement was reached.

Many comments were written alongside a specific QL questionnaire item or subscale, which helped determine the appropriate theme. The patient’s exact wording provided the over-riding factor in guiding categorisation, for example *”Most of my concerns are caused by work rather than my illness and treatment"*  would be categorised as a work-related comment.

Multiple comments from an individual patient were included in more than one category. For example: *“My life has changed drastically since I was diagnosed but it had nothing to do with my breast cancer…. Firstly I moved home, then my mother died, followed by my husband’s death and finally with eye surgery and a diagnosis of arthritis……”* In this case the comments were allocated to three themes, ‘life events’, ‘chronic medical/mobility’ and ‘surgery’.

Comments of a practical nature (such as apologies for late return of the questionnaire) were not coded. No assumptions were made about a concern or symptom having an enduring effect time (e.g. possible chronic disability or permanent functional difficulty) and no weighting was given to any of the proffered explanatory comments received.

**Results**

Overall, 22% of women (482/2181) with evaluable data in the quality of life sub study proffered written comments in their booklets, or included letters, at least once between baseline and two years follow-up., increasing over time. The number of women making comments at baseline, 6, 12 and 24 months was 97/2181 (4.4%), 180/2046 (8.8%), 175/1985 (8.8%) and 236/1888 (12.5%) respectively. Characteristics of the overall trial cohort and the subgroup commenting over this time are shown in Table A.1 of this appendix. This shows broad comparability with the overall trial cohort. Differences observed were generally small but statistical testing was not deemed reliable given the unselected nature of the women making comments.

Nine themes were created (Table A.2 in this appendix) and were relatively straightforward to identify; examples are shown in this appendix. Physical functioning and chronic medical problems were the most frequently used themes but psychological problems and life events / family problems were also often reported. These accounted for the majority of contextual life difficulties reported, many of which are not reflected in the self-report QL measures. Women frequently wrote alongside the relevant questionnaire item or PROM domain indicating that their rating reflected a non-cancer / non-treatment cause and wanted us to be aware of that.

Questions in the health economics section of the QL booklet concerning work status at one year follow-up showed that 77% women (834/1107) had returned to work at this point. Work status was unknown for 213 women and 580 were retired at baseline, but of those not returning to work 70 (50% of non-returnees) made explanatory comments, such as ‘ill health’, ‘made redundant’, ‘retired early’, or ‘business closed’.

To investigate the women’s concerns that their contextual problems may negatively affect associated QL ratings, we compared the EORTC functional domains and HADS Anxiety and Depression subscale scores for women commenting at the same assessment point with those of women who did not comment on that occasion. These showed differences in the expected direction, that is, worse scores for women commenting on their concerns or contextual difficulties, for all variables (Data not shown). However, as the women commenting were a self-selected group, this was not deemed a reliable basis for inferring QL outcomes.

**Implications**

Women’s spontaneous comments, written in a naturalistic, unsolicited setting, highlighted concerns and situations in their lives that have been widely shown to affect the quality of life of breast cancer patients. They reflect the importance of the social context in the clinical trials setting and are relevant to the provision of support and holistic care. Such findings, if consistent in other trial patients, might encourage a more cautious interpretation of secondary endpoints in clinical trials such as START.

Therefore a decision was made to invite all patients to contribute comments at the final 5-year assessment, saying “*If you wish to write comments about any concerns, health problems or events which you think might affect the answers you give in the QL booklet, please do so”.* This new analysis of invited comments is fully reported in the main paper.

**References**

Pope C Ziebland S, Mays N. Qualitative research in healthcare: Analysing qualitative data. BMJ 2000; 320: 7227. 114

**Themes derived from proffered comments (0-24 months follow-up), with examples**

**Physical Functioning**

‘I had difficulty completing the form as not sure which questions refer to breast cancer and which to general health. I have a crushed vertebra, which causes me considerable pain and discomfort. I suffer no pain from radiotherapy’

‘I should stress that all slowness and extra time taken on simple tasks stems from a worsening hip condition. I am still waiting for a hip operation when I hope my mobility will be better next time you send me questions’

**Chronic medical problems**

‘I may be wrong but I feel any health symptoms I have are more to do with my heart problem, e.g. walking, breathing during strenuous activities, than my breast lumpectomy. I hope you will allow for this in my answers’

‘Because I suffer from a mild form of MS my state of health depends on a level of fatigue. I don’t think the cancer treatment has changed anything’

**Breast related problems**

‘After my operation, I came home and a nurse told me that my breast was infected, it really hurt and I had to return to have my breast drained, a very painful experience’

‘I have been unable to answer some of the questions properly as a cancer was found in the other breast, a different type of cancer, it was removed and I now receive chemotherapy. I have no discomfort from the last cancer nor the radiotherapy’

**Systemic treatment effects**

‘I have come off tamoxifen as I put on loads of weight and my eyesight has been affected, also the hot flushes have been horrendous. I just wanted to make it clear why I’m dissatisfied with my appearance and why sometimes I’m a bit low’

‘Please note that a lot of my replies are due to tiredness caused by difficulty sleeping. This is likely to be caused by the tamoxifen’

**Surgery**

‘A lot of my answers are affected by the fact that I had major heart surgery to have a mechanical heart valve replacement only 10 weeks before I had a mastectomy’

‘Problems relate to hysterectomy operation. Still recovering’

**Acute and transient health problems**

‘The past week has definitely not been my ‘best week’. I went down with a heavy cold and awful cough’

‘Recovering from shingles which was very painful and my answers reflect this’

**Psychological problems**

‘I have been taking prozac since diagnosis and feel that this has affected my mood. I have experienced a change of mood since stopping HRT and commencing Tamoxifen with a return of menopausal symptoms’

‘I have been feeling anxious and my GP has prescribed Seroxat tablets which I have been told will take a month or so to have an effect. So far I have been taking them for 2 weeks so hopefully when I complete the next booklet I will be feeling fine’.

**Life events**

‘My husband left me 2 weeks after surgery (not connected to surgery) and during my chemotherapy I got divorced….’

‘My mother has also recently learnt that two of her cousins (on her mother’s side of the family) have also had breast cancer…’

**Job problems**

‘I was extremely stressed by bullying in my work place before I was diagnosed. I have not yet returned to work but I am still emotionally affected by visits and phone calls from my employers Most of my concerns are caused by work rather than my illness and treatment’

‘I returned to work for a period of two months only, before being signed off sick again… I eventually had to let the job go, as recommended by my doctor’

**More than one theme**

‘My life has changed drastically since I was diagnosed with breast cancer in 2000 but nothing to do with the breast cancer. Firstly I moved home, then my mother died, followed by my husband’s death, and finally with eye surgery, and a diagnosis of arthritis for which I take strong painkillers’

‘I feel tense or ‘wound up’ because I am looking after my 96 year old Aunt. I look forward with enjoyment to things rather less than I used to, because of my arthritis. Also my husband has prostate cancer’

**Table A.1: Characteristics of patients who proffered comments at 2 years and for the overall QL cohort enrolled in the START Trials Quality of Life study**

|  | **Overall Cohort**  **N= 2208 (%)** | **Proffered comments subgroup (0-2 years)**  **N= 482 (%)** |
| --- | --- | --- |
| Age at baseline (years): mean (SD) [range] | 56.9 (10.4)  [26-86] | 57.6 (10.6)  [28-81] |
| **Highest level of education achieved^1^** | | |
| School certificate / O-level / GCSE / NVQ or equivalent | 528/1899 (27.8) | 114/438 (26.0) |
| A-Level / HND or equivalent | 115/1899 (6.1) | 31/438 (7.1) |
| Degree, post-graduate or professional qualification | 439/1899 (23.1) | 124/438 (28.3) |
| None of the above | 725/1899 (38.2) | 151/438 (34.5) |
| Unknown – not completed on form | 92/1899 (4.8) | 18/438 (4.1) |
| **Employment prior to diagnosis^1^** | | |
| Full-time | 540/1899 (28.4) | 130/438 (29.7) |
| Part-time | 443/1899 (23.3) | 87/438 (19.9) |
| Self-employed | 132/1899 (7.0) | 33/438 (7.5) |
| Retired or household work | 1095/1899 (57.7) | 263/438 (60.0) |
| Other (voluntary, job-seeking, student, sick leave) | 281/1899 (14.8) | 79/438 (18.0) |
| Unknown – not completed on form | 41/1899 (2.2) | 6/438 (1.4) |
| **Type of surgery** | | |
| Breast-conserving surgery | 1831 (82.9) | 392 (81.3) |
| Mastectomy | 377 (17.1) | 90 (18.7) |
| **Adjuvant systemic therapy** | | |
| None | 128 (5.8) | 32 (6.6) |
| Chemotherapy alone | 224 (10.1) | 50 (10.4) |
| Tamoxifen alone | 1266 (57.3) | 267 (55.4) |
| Chemotherapy & Tamoxifen | 537 (24.3) | 118 (24.5) |
| Other | 42 (1.9) | 14 (2.9) |
| Unknown | 11 (0.5) | 1 (0.2) |
| Global heath/QoL during past week at baseline^2^: median (IQR) | 66.7 (56.2-83.3) | 66.7 (50.0-83.3) |

SD= standard deviation, IQR=interquartile range

^1^ Education and employment data collected at 1 year after randomisation, so not available for all participants (1899 and 438 1-year forms completed for overall cohort and 2-year proffered comments group respectively). Women could tick more than one employment category, hence percentages add up to >100%.

^2^ Subscale from EORTC QLQ-C30; score ranges from 0-100, with *higher* scores indicating *better* global health / quality of life.

**Table A.2: Themes derived from women’s proffered comments showing most frequent component topics, received between baseline and 2 years’ follow-up in the START Trials**

| **Physical functioning:** Arthritis (rheumatoid and osteoarthritis) fractures or falls, back or joint pain, fibromyalgia, muscle pain or injury, mobility problems |
| --- |
| **Chronic medical problems:**  Asthma, breathlessness, diabetes, heart disease, hypertension, skin rashes, drug reactions (excluding Tamoxifen or other cancer therapies), chronic conditions e.g. MS. Migraine, Parkinson’s disease |
| **Breast and related problems:**  Arm lymphoedema*,* cancer recurrence in the breast, radiotherapy effects on the breast |
| **Systemic treatment side effects:**  Weight gain, hot sweats ‘tamoxifen side effects’ |
| **Surgery (excluding breast cancer related):**  Gynaecological, dental, or other surgery*.* |
| **Acute or transient health problems:**  Colds, viral infection, ‘stomach bug’ |
| **Psychological problems:**  Depression or taking antidepressant medication, history of chronic mental illness (e.g. schizophrenia) anxiety disorders, panic attacks |
| **Life events or family problems:**  Bereavement, husband’s illness, house move |
| **Job problems:^1^**  Redundancy, early retirement, job loss and other job related issues |

^1^Only collected in years 1 and 2

**Appendix B: Themes & Examples of Comments made at 5 Years in the START Trials**

***Instructions given:***

*Written at the top of a blank page in the 5-year Quality of Life booklet: “Please feel free to add your own comments. We will be very pleased to have them”*

A letter to every patient was enclosed with the 5-year QL Booklet, saying: *‘If you wish to write comments about any concerns, health problems or events which you think might affect the answers you give in the QL booklet, please do so. You will find a blank sheet of paper at the end of the booklet. This is entirely voluntary, but please feel free to write as little or as much as you want. Your comments will, of course, remain anonymous and confidential but they may be used to help inform doctors and nurses about the results of the study in publications and we will ensure you cannot be identified by them.’*

**Chronic Medical problems and physical functioning**

‘Any negative replies I have given do not refer to my breast cancer treatment, but totally unconnected medical problems’

‘Osteoporosis and IBS cause pain in my lower body. Not worried about breast cancer and doing OK’

‘I suffer from diabetes, heart disease and IBS, this will have affected answers’

**Breast and related problems**

‘My main problems have been about the use of my right arm, post axillary clearance and this does limit my use of that arm for carrying bags and swimming, rowing etc’

‘I have had further recurrences of cancer. I found lumps under my other arm, all were cancerous. Had a second mastectomy. Feel as if living with a time bomb’

‘I feel self-conscious about my breast as red lines around armpit from RT and black ring around nipple’

‘The skin problems with my affected breast are chiefly the noticeable outline of the radiotherapy area, which I understood would disappear in 6 to 12 months after cessations of treatment, but which now looks as if it will be a permanent feature’

**Systemic treatment side effects**

‘I was on Tamoxifen for 2years and Arimidex for 3 years…. these have made my life extremely uncomfortable and none of the remedies worked. This had a huge negative effect on sleeping, sex, weight-gain, morale and self-esteem as well as general life.....However the quality of life, even after cancer should be treated with equal importance and one should not be made to feel ‘lucky to be alive!’

‘The on-going problem of hot flushes has severely restricted my social life and also relationships with others’

‘I feel cheated that the drugs took away all sexual desire.... Now at 57 my sex life will never return. But mentally I feel I should be enjoying sex’

**Surgery (excluding breast cancer related surgery) and Hospital Admissions**

‘I had a knee replacement in July. Had mild ulcerate colitis in Oct and admitted to hospital last week, this affects answers’

‘Until this year felt very well, active and looking forward to a holiday of a lifetime. Then I was rushed to Hospital for keyhole surgery and they found an ovarian cyst. Been off work since’

‘I have recently been in car accident and suffered a fractured sternum and whiplash. At present unable to carry out normal duties at home and work’

**Acute or transient health problems**

‘Suffering from double vision following eye tests. Awaiting results which is giving me anxiety..’

‘My health and quality of life affected by cough and cold virus also sore throat which has kept me awake at night’

‘These answers are not a true reflection of my breast cancer. I have had flu for the past week and am still recovering’

**Psychological problems**

‘Carer for husband for 10 yrs. Mood swings due to his illness’

‘Felt quite low for some time, could be due to a lot of trouble at home with some of the family and nothing to do with my cancer…’

‘My tension is nothing to do with my breast cancer. It is to do with my husband who wishes to spend retirement abroad. I don't wish to leave this country, friends & family. Causing tension and anxiety’

**Life Events: General and Family problems**

‘Just lost youngest daughter (39 years) to cancer’

‘Since RT I have moved house, lost my husband, moved again, started part time business and met a new partner. Much of my anxiety or worry has been attributed to these life experiences’

‘Widowed last year, which has affected my answers’

**Life Events: Job problems:**

‘After diagnosis I lost my job. I believe that there was a connection and that led to my depression and anxiety’

‘I am tired a lot of time and feel unable to work and pursue leisure activities’

‘I am uncertain about the future in relation to work. The saying "the misery of certainty is far worse than the certainty of misery’

**Other cancers**

‘I now have bowel cancer’

‘I have been diagnosed with cancer of the ovary’

‘I have acute myeloid leukaemia’

**Aspects of Care (negative)**

‘Delay in initial treatment, GP did not refer me for a year. Feelings of anger added to distress & anxiety’

‘I’ve never seen my consultant and felt that everything was rushed and would have liked to speak to a breast care nurse about the alternative treatments….’

‘Not enough support given when treatment is finished. More help should be given’

**Family History of Cancer**

**‘**I am v anxious and depressed because cancer is rife in my family’

‘Work part-time, I am tired and anxious as family history of breast /ovarian cancer

‘My sister died 10 yrs after diagnosis with ovarian cancer so it’s at the back of my mind’

**Effects of Ageing**

‘I find it difficult to distinguish whether any minor problems are due to cancer treatment or to getting older e.g. occasional forgetfulness, weight gain’

‘My aches & pains are due to age rather than treatment. Delighted with the team that took care of me. Health at my age is v good’

‘My health problems are to do with age and arthritis’

**Future concerns: Cancer related worries**

‘In the last 6 months, 2 of my friends have died from breast cancer and another friend is in a hospice........ Mostly I consider myself to be well and cured of the illness, but from time to time I remember what I’ve been through and wonder what the future holds especially when I see my friends suffering’

‘Even now 5 years on, I am very worried and sometimes I can’t sleep properly, it is always on my mind’

‘When I stopped Tamoxifen I felt very frightened, and worried that the cancer would re-occur. I felt as if a safety net had been pulled from under me’

**Positive Comments:**

**Good Aspects of Care**

‘Thanks for care and support during the 5 years’

‘Been interesting doing the trial. Made me think about my health and well-being’

‘Cancer turned out to be blessing’

‘Pleased to have been part of the trial and hope it helps in some way’

**Good Recovery from Cancer**

**‘**I remain positive, have an excellent social circle and enjoy my life. Cancer has given me a very positive outlook on life, I realise the value of friendship, grabbing opportunities and living each day to the full’

‘I’ve changed my outlook to life – I take this as a wake-up call to appreciate life and to look after myself more carefully in terms of food and exercise. Through my treatment I was always worrying “what if" in case it came back. Now I've been discharged I feel a sense of relief to get on with life’

‘I’ve tried to look into more spiritual and positive things in life and get on to life in a positive way’

**Personal Support (friends, family, GP, religion)**

‘Everyone has been so kind and happy to answer any questions and fears I had. I am a Christian and feel that my faith has played a vital part in my recovery’

‘I have a positive attitude to most things and with the help of a loving family, great friends and neighbours with plenty of interests inside the home and outside’

‘I have had a positive attitude the whole time and a lot of help from family, quite sure that helped. Plus most important of all I have real faith in God, in a very quiet way’

**Positive Life Events**

‘Recently completed a degree which has helped with my feelings of wellbeing & self-esteem. Each day feel stronger and more alive’

‘Breast cancer has not held me back. 6 months walking in India’

‘I have now qualified as a teacher, a new life and partner’

**Mixed Comments (not mentioned in table)**

‘Reconstruction (flap) has made a big difference, helped mentally. Felt depressed for other factors unrelated to health. Hot flushes improved and now stopped tam and hopefully lose some weight. Good thing about breast cancer has been the focus on important things in life’

‘Have arthritis therefore trouble with a long walk. Have diabetes so feel nauseas at times. Treated for depression with Prozac for 9mnths. Now feel great look forward to future and consider myself very fortunate’

‘Suffer slightly from depression 3 months after radiotherapy onwards and continuing. Receiving anti-depressants for anxiety stress and headaches. Information from the team was excellent. Completing the questionnaires has helped in appreciating how far I’ve progressed in 5 years’

**Comments about commenting**

‘I have a few symptoms unrelated to breast cancer. Facial nerve spasm affects my eyes. Q does not give an opportunity to say if any aspects of current health are felt to be linked with breast cancer. ‘

‘Very concerned no questions asking other factors in my life. One problem was telling already stressed family members of my health problems - made my life even more difficult’
